# PEG/Dextran Double Layer Influences Fe Ion Release and Colloidal Stability of Iron Oxide Nanoparticles

**DOI:** 10.1038/s41598-018-22644-8

**Published:** 2018-03-09

**Authors:** M. Rezaa Mohammadi, Andrey V. Malkovskiy, Preetha Jothimuthu, Kwang-Min Kim, Mansi Parekh, Mohammed Inayathullah, Yan Zhuge, Jayakumar Rajadas

**Affiliations:** 1 Biomaterials and Advanced Drug Delivery Laboratory, Stanford University School of Medicine, Stanford, CA 94305 USA; 2 Department of Radiology, Stanford University School of Medicine, Stanford, CA 94305 USA; 3 Department of Bioengineering and Therapeutic Sciences, University of California San Francisco School of Pharmacy, San Francisco, CA 94158 USA

## Abstract

Despite preliminary confidence on biosafety of polymer coated iron oxide nanoparticles (SPIONs), toxicity concerns have hampered their clinical translation. SPIONs toxicity is known to be due to catalytic activity of their surface and release of toxic Fe ions originating from the core biodegradation, leading to the generation of reactive oxygen species (ROS). Here, we hypothesized that a double-layer polymeric corona comprising of dextran as an interior, and polyethylene glycol (PEG) as an exterior layer better shields the core SPIONs. We found that ROS generation was cell specific and depended on SPIONs concentration, although it was reduced by sufficient PEG immobilization or 100 µM deferoxamine. 24 h following injection, PEGylated samples showed reduction of biodistribution in liver, heterogenous biodistribution profile in spleen, and no influence on NPs blood retention. Sufficient surface masking or administration of deferoxamine could be beneficial strategies in designing and clinical translation of future biomedical SPIONs.

## Introduction

Superparamagnetic iron oxide nanoparticles (SPIONs) have sparked commercial motivations in biomedical industry including cell labeling^1^, iron deficiency anemia^2^, targeted drug delivery^3,4^, magnetic resonance imaging^5^ and immunotherapy^6^. Commercial SPIONs generally possess a physiosorbed dextran layer around their core. Unfortunately, these nanoparticles (NPs) have demonstrated toxicity and side effects in clinical trials^7^. For instance, 23% of patients receiving Ferumoxtran-10 (a dextran coated SPION) were reported to experience side effects^8^. Another main issue is that dextran undergoes biodegradation via various dextranases found in many tissues^9^. Unfortunately, replacing dextran with other polymeric coatings did not show promising results as well. For instance, commercial polyethylene glycol (PEG) modified SPIONs, such as Clariscan™ (NC100150), demonstrated toxicity^10^.

Upon cell entry, biodegradation of SPIONs in endosomal environment generates oxidative stress which is known to be a major source of SPIONs toxicity^11^. It has been demonstrated that endosomal biodegradation of SPIONs leads to formation of ferric ions stored in ferritin^12^. These ions are then reduced to ferrous ions via reductases, leading to formation of oxidative stress^13^. It is also reported that dextran coated SPIONs are highly prone to biodegradation^14^. In addition to biodegradation, unprotected surface of SPIONs mediate the generation of hydroxyl radical through catalytic activity. Voinov *et al*. demonstrated that catalytic activity of the γ-Fe_2_O_3_ and Fe_3_O_4_ NPs is ascribed to the reactions at the NPs’ surface^15^. Furthermore, they reported that the catalytic centers on the NP surface were at least 50-fold more effective in the production of the OH^•^ radical than the dissolved Fe^2+^ ions.

Even though it is believed that a masked surface of SPIONs could eliminate their adverse *in vivo* interactions^11^, few studies have focused on designing a polymeric corona to better mask the SPIONs surface. Moreover, one required aspect of bringing nanomedicine to patients is to fully realize the *in vivo* fate and biodistribution of NPs^16^.

To better mask the surface of dextran coated SPIONs, we sought to formulate an additional coating around them. We hypothesized that a nanocomposite comprising of a second protective PEG layer around the dextran coated SPIONs may efficiently block the cytotoxic Fe ion leakage from the core or could limit the access of molecules to the catalytic surface of SPIONs. Designed NPs is this study demonstrated that physisorption of sufficient amount of PEG layer around dextran coated SPIONs not only improves their colloidal stability, but also reduces the reactive oxygen species (ROS) generation compared with dextran coated SPIONs. We also assessed the influence of PEG/dextran double layer on biodistribution and pharmacokinetics of NPs, and results confirmed the proper surface coverage of SPIONs results in greater circulation time.

## Results

In this study, SPIONs were coated with a double layer of dextran (interior layer) and PEG (exterior layer). In this respect, three samples were formulated i.e. (1) PDS0: a dextran coated SPIONs, (2) PDS1: a physically PEGylated PDS0 with 1:1 PEG:SPION weight ratio, and (3) PDS8: a physically PEGylated PDS0 with 8:1 PEG:SPION weight ratio. Following by detailed physicochemical evaluations, NPs were assessed for ROS generation in 3 cell lines through 4 h of co-incubation. Deferoxamine, an Fe chelator, was also employed as a control for Fe ion mediated ROS generation. Finally, biodistribution and pharmacokinetics of samples were evaluated within 24 h post injection of 2 mg/ml of samples.

### Physicochemical characterization

**Figure 1:**
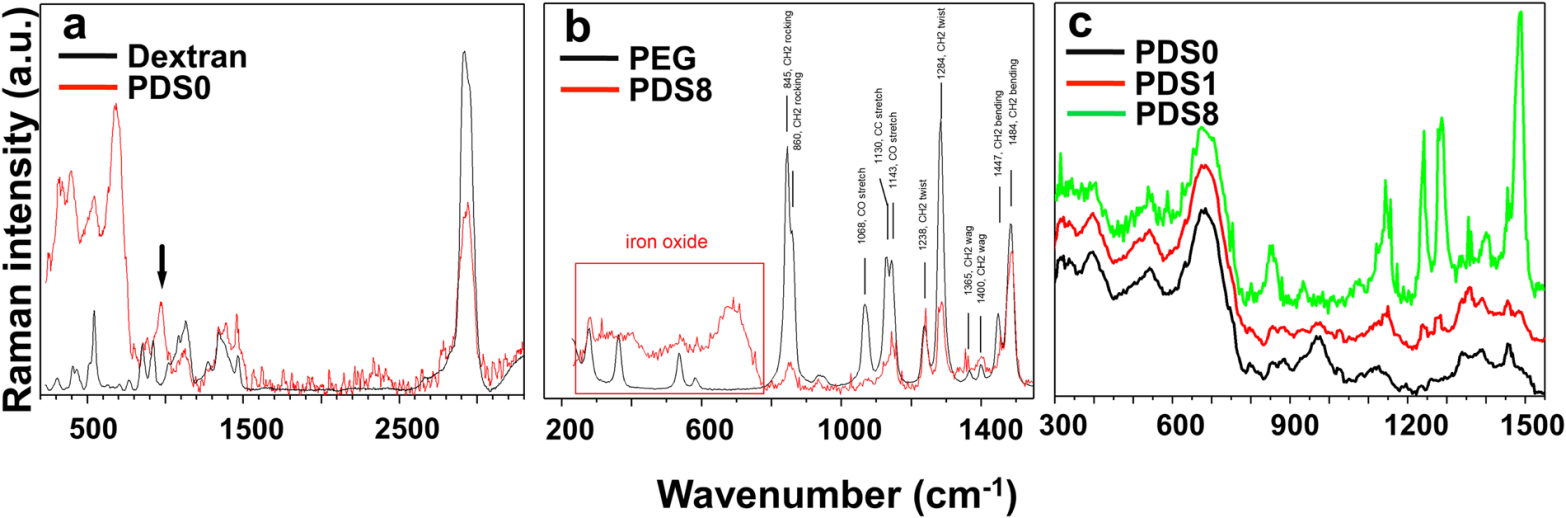
Raman spectra of (**a**) dextran and PDS0, (**b**) PEG and PDS8, and (**c**) PDS0, PDS1 and PDS8, normalized to iron oxide bands. C-O-H bending vibration of dextran is marked with a black arrow. Characteristic bands for PEG and PDS8 in the fingerprint region have been identified and summarized in Supplementary Table 1.
   Fig. 1b shows Raman spectra of PEG and PDS8 and band assignments for various PEG molecular vibrations, indicating the presence of PEG around dextran coated SPIONs (more descriptive information is provided in Supplementary Table 1**)**. In the row of PDS0-PDS1-PDS8 (Fig. 1c), C-O-H bending vibration gradually disappears, as dextran constitutes an even smaller fraction of the complex particle. At the same time, PEG vibrations become more and more pronounced. Interestingly, the C-O stretch of PDS8 is less than 8 times higher (only about 2 times higher) than that of PDS1, indicating that bonding between PEG and dextran-coated SPIONS is most effective for PDS1, while PDS8 has longer sections of PEG without attachments. This is further confirmed by strong CH_2_ bands for PDS8 (860, 1238, 1284 and 1484 cm^−1^) that can be barely observed for PDS1. Apparently, these vibrations are heavily restricted in the PEG segments confined between SPIONs. These bands are even higher for pure PEG, where there are no such restrictions.
- *Fourier transform infrared spectroscopy (FTIR)*
  To further investigate the physical bond formation between PEG and dextran, FTIR spectroscopy was conducted. Fig. S2-a shows the characteristic bands of PEG, which the –C–O–C– ether stretch band and the vibration band (antisymmetric stretch) are appeared at 1101.1 cm^−1^ and 1349.4 cm^−1^, respectively^22^. In addition, the absorption bands at 1281.3 cm^−1^ and 1468.8 cm^−1^ attribute to the vibration of –CH_2_^23^. Fig. S2-b shows the FTIR bands for bare SPIONs to ease the comparison. In the broad peak near 3450 cm^−1^ belongs to attached hydroxyl groups^22^. PDS1 spectrum (Fig. S2-c) comprises the main absorbance of ether stretch band at 1098.9 cm^−1^ and –CH_2_ vibrational band at 1275 cm^−1^ and 1459 cm^−1^. Fig. S2-d shows PDS8 spectrum demonstrates the main absorbance of ether stretch band at 1097.8 cm^−1^ and –CH_2_ vibrational band at 1273 cm^−1^ and 1461 cm^−1^. These spectra verify the presence of PEG on the surface of NPs. However, the characteristic absorbance peaks of PEG show a slight shift to lower frequencies (red shift) due to changing the environment of PEG^22^ as well as hydrogen bonding between PEG and dextran^24^.
  Another important point is that the wavenumber of δO–H deformation modes of dextran hydroxyl group^25^ for PDS0, PDS1, and PDS8 are 1620 cm^−1^, 1634.8 cm^−1^, and 1634.8 cm^−1^, respectively, which suggests dextran-PEG interaction in PDS1 and PDS8 samples. Besides, –CH_2_ transmittance band for samples PDS0, and PDS1 are 2920 cm^−1^, 2912 cm^−1^, and 2908 cm^−1^, respectively. This redshift is likely due to lengthening of C–H bond^24^ caused by more interaction between PEG and dextran. These results in their totality are likely to reveal that hydrogen bonds have been formed between the PEG and dextran in PEGylated samples.
- *Thermogravimetric analysis (TGA)*
  Weight loss curves are presented in Fig. S3. The first stage of weight loss that onsets from early stage up to 180 °C is due to the evaporation of water molecules. The second stage starting from 220 °C to 400 °C is attributed to the dextran and PEG degradation^26^. The incline in weight loss is the proof of increase in coating density around the surface of SPIONs. The weight loss boosts from 15% in PDS1 to 71% for PDS8, demonstrating the higher amount of immobilized PEG around PDS8.
- *Vibrating sample magnetometer (VSM)*
  Magnetic properties were obtained via VSM at 300 K. Fig. S4 shows magnetic response, and the lack of hysteresis loop indicates the superparamagnetic nature of all the samples. M_s_ values slightly decreased by addition of coating and the corresponding Ms values are provided in Supplementary Table 2. To correctly compare saturation magnetization (M_s_) values, the M_s_ values were normalized to the mass of iron oxide^27^. It is generally believed that the M_s_ reduction of coated NPs is due to surface protection of the coating agents^28,29^. Further, it is established that the polymeric corona (shell) interacts with the surface atoms of the magnetic particles (core), leading to formation of a magnetically disordered layer, which reduces the magnetic phase^17^.
- *Atomic Force Microscopy*
  To study the size, morphology, and the polymeric coating around SPIONs, we conducted AFM analysis. Fig. 2 demonstrates height and phase images associated with each sample. PDS0 shows nanoscale particles, but at the same time large (micron-scale) aggregates are also observable (Fig. 2 and Fig. S5). Following by adding PEG, we could not observe these aggregates, but smaller-scale clusters appeared following by PEGylation in sample PDS8. Sample PDS1, however, showed both large aggregates and smaller clusters. To further validate these observations, electron microscopy was conducted.

- *X-Ray Diffraction (XRD)*
  Fig. S1 shows the XRD pattern of PDS0, PDS1, and PDS8. Magnetic particles in this study possess similar characteristic peaks of Fe_3_O_4_ as reported elsewhere^17^, and are well-matched with the magnetite (Fe_3_O_4_) diffraction peaks (JCPDS card no. 19-0629), confirming their inverse spinel structure. Greater PEG/SPIONs weight ratios culminate in depression of diffraction intensity, indicating higher amount of PEG on SPIONs.
- *Raman Spectroscopy*
  To study the presence of the PEG/dextran double layer coating around SPIONs, we performed Raman spectroscopy for dried PDS0, PDS1, PDS8, as well as for pure dextran and PEG. Raman spectra of dextran^18,19^ and PEG^20^ agree well with those reported in literature for the whole range of wavenumbers probed.
  Raman spectrum of PDS0 below 800 cm^−1^ (Fig. 1a) is overwhelmed by the iron oxide bands^21^, however there are clearly dextran bands at higher wavenumbers. Most peak positions stayed the same with the exception of the C-O-H bending vibration of dextran^18^ at 916 cm^−1^, which shifts to 972 cm^−1^ (marked by black arrow on Fig. 1a). This indicates that almost all COH groups have reacted to produce dextran-SPION linkages.

**Figure 2:**
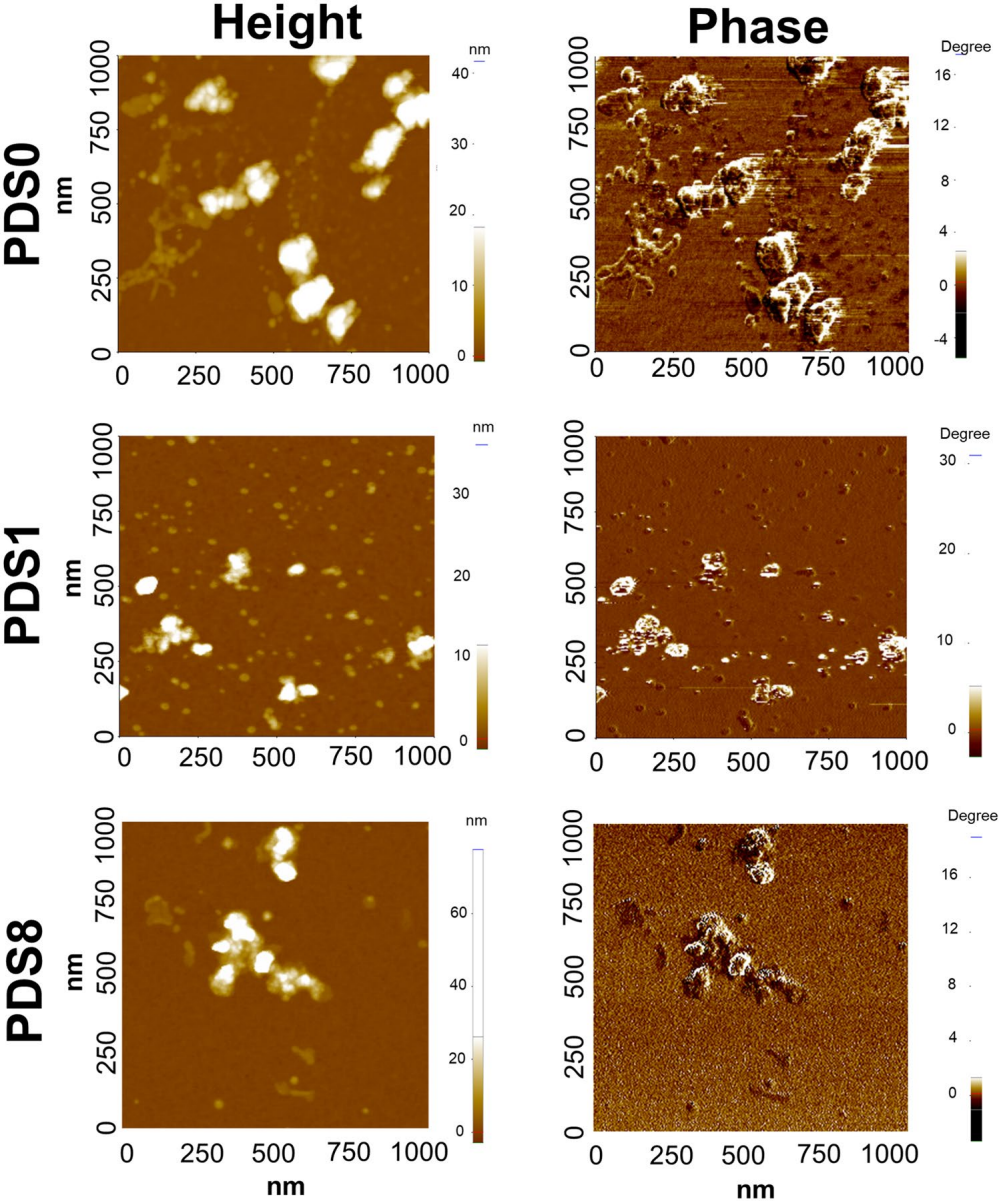
Analysis of NPs with one square micron AFM maps of SPION nanoparticles. Figures in the left and right represent the height and phase images, respectively. The dextran coated particles (PDS0) are forming large aggregates as well as individual NPs, and PEGylation leads to less large-scale aggregation, and smaller-scale clusters. The topography maps for PDS1 and PDS8 show the formation of complex aggregates of NPs where individual particles are connected by PEG.
- *Electron Microscopy analysis*

**Figure 3:**
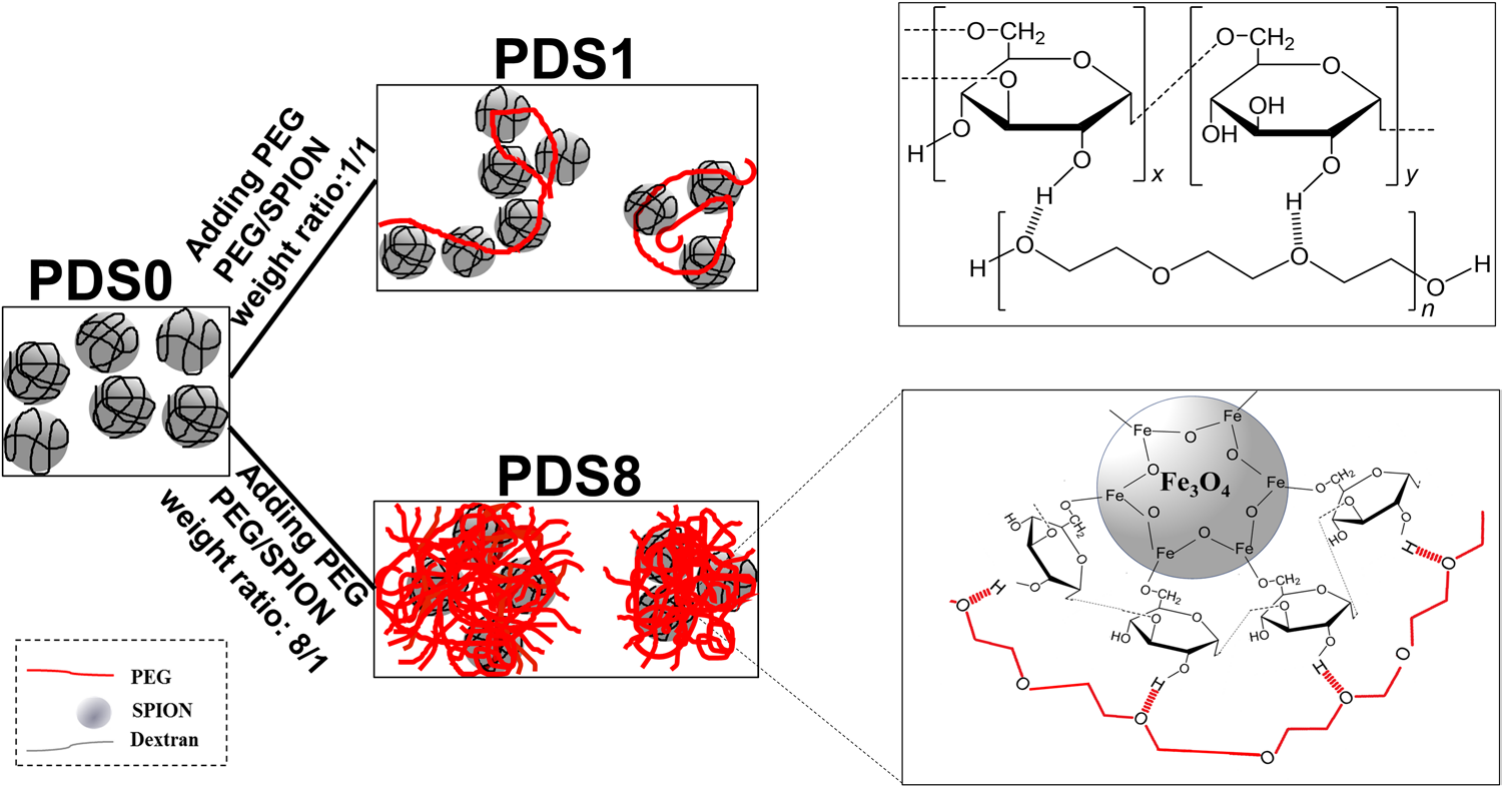
Schematic representation of corona formation around NPs, and the suggested physical bonding between PEG and dextran (dash lines between the PEG and dextran represent Hydrogen bonding).

Similar to the AFM observations, both scanning and transmission electron microscopy analyses demonstrated that individual NPs as well as large aggregates are present in PDS0 (Fig. S6). By addition of PEG, large aggregates were not observable, however, small colonies were formed (sample PDS8). From the physicochemical characterization of NPs in this study we proposed a model in Fig. 3, which demonstrates the schematic representation of possible polymeric corona and the bonding between components. It should be noted that PDS0 are mostly separate but also with some clumps formed by capillary forces acting on the drying sample. When dynamic light scattering (DLS) results are combined with AFM and electron microscopy data (Supplementary Table 3), it is apparent that hydrodynamic size increases with PEGylation ratio, suggesting that large artifacts in PDS0 sample are drying artifacts.

### Colloidal Stability Assessment of NPs

**Figure 4:**
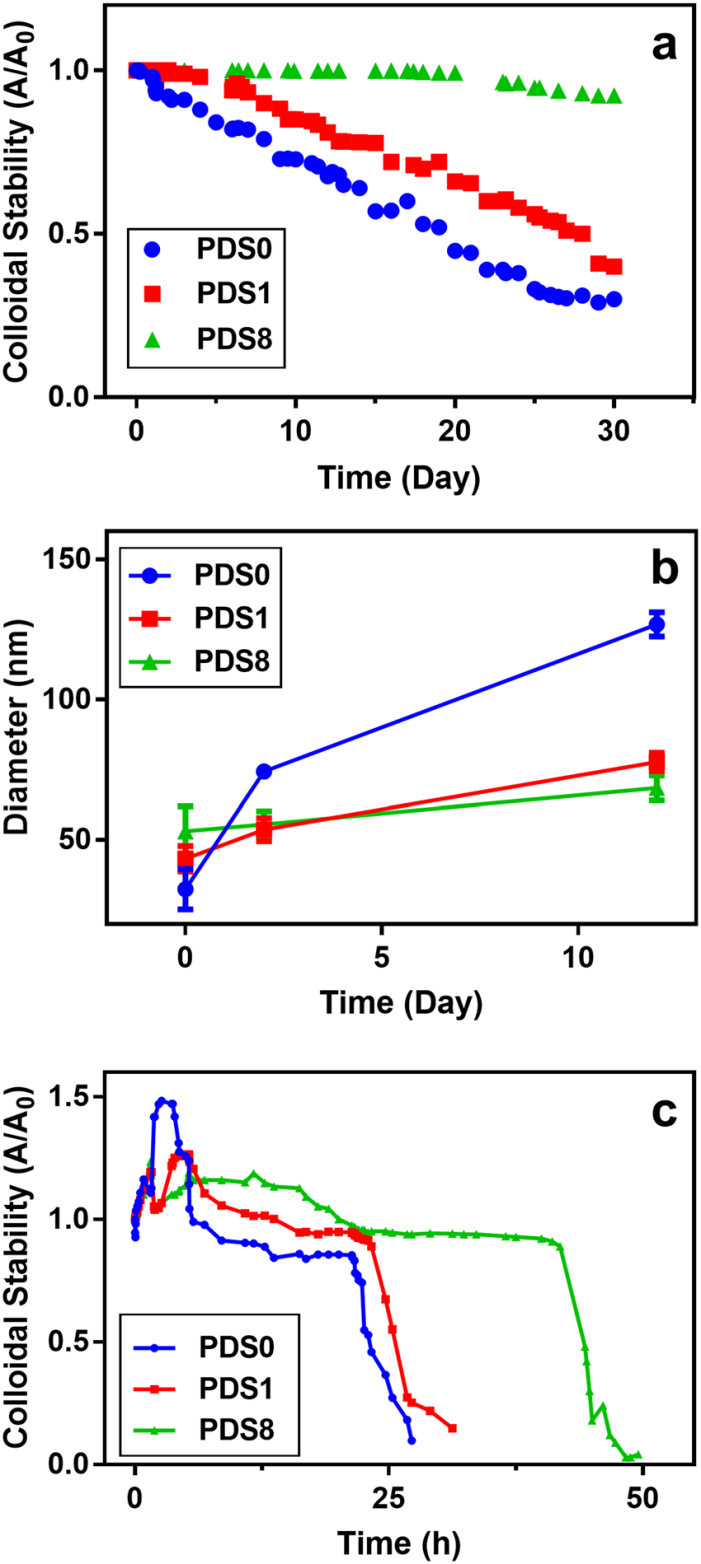
Colloidal Stability assessment of NPs in DI H_2_O and cell culture media. (**a**) The absorbance of the NPs was measured at different time points and normalized over initial absorbance. Relative absorbance of samples, and (**b**) particle hydrodynamic size stability over time are measured in DI H_2_O (n = 3) to evaluate the colloidal stability. (**c**) Relative absorbance of samples was also assessed in RPMI 1640 + 10% FBS to simulate their colloidal stability in physiological media. Samples for colloidal stability experiment in cell culture were kept in incubator during the time of experiment. Error bars indicate mean ± SD (n = 3).

We measured the optical absorbance of NPs which is a function of sedimentation of agglomerated particles caused by gravitational forces^30^. As shown in Fig. 4a, the relative absorbance of samples PDS0 to PDS8 in DI-H_2_O is reported over a period of a month at room temperature. Results indicate that PDS8 possesses greatest colloidal stability over the experiment period, as the relative absorbance decreased by 9% after 30 days, whereas for PDS0 the relative absorbance reduced by 9% in the third day of experiment and decreased by 75% after 30 days. It should be noted that no sensible change in UV absorption was observed for the PDS8 sample up to 17 days after initial date of experiment. Noteworthy to mention that samples maintained their colloidal stability at 4 °C for 6 months.

Early settling down of PDS0 sample was observed as A/A_0_ decreases within the second day of experiment. However, the physical appearance was the same during the first ten days, with no visible sedimentation. The sedimentation rate decreases as the PEG/SPION weight ratio reaches its critical concentration in PDS8 sample. While for the PDS1 sample the first detectable decrease in absorbance occurs as early as the second day of experiment, settling down was not observed for the PDS8 sample up to 20 about days. One reason that the exterior PEG layer enhances the colloidal stability is due to its flexible nature. PEG has highly flexible hydrocarbon chains, with many plausible conformations. This behavior leads to formation of a conformational “cloud” around the NPs^31^.

DLS study was performed to further analyze the hydrodynamic size variations over time (Fig. 4b). At day 0, PDS0 had the least hydrodynamic size (32.4 ± 8.1 nm). The hydrodynamic size profiles of PDS1 and PDS8 match (46.2 ± 4.9 and 53.0 ± 7.5), although after 10 days they start to deviate.

The growth rate of hydrodynamic diameter over time could be considered as colloidal stability measurement. For instance, size of PDS0 demonstrates the fastest growth rate over other samples, while PDS8 manifests the lowest rate of hydrodynamic size growth. These results indicate that incorporation of a second PEG layer around the initial dextran layer enhances the colloidal stability of particles, even though the bonding between the layers are purely physical. Another point is that the amount of PEG layer dictates the hydrodynamic size, and colloidal behavior of samples.

To mimic the *in vivo* colloidal behavior of NPs, relative UV absorbance of NPs was measured in cell culture medium (CCM). Fig. 4c illustrates the relative absorbance of samples in RPMI 1640 medium + 10% FBS. At early stage of colloidal stability, UV absorption behavior of PDS0 manifested a long-range elevation (hook effect) in absorption (Fig. 4c), which may be explained by the prevalence of dimer formation^30^. This elevation that initiates following by 1.5 h incorporation of PDS0 in CCM, indicates SPIONs coated with dextran tend to coagulate rapidly. As the PEG layer added, coagulation kinetic changes significantly. For instance, the sudden coagulation onset of 1.5 h for PDS0 shifts to about 4 h and 5 h for PDS1 and PDS8, respectively (Fig. 4c). This observation is consistent with a previous study, where EDC/NHS chemistry was employed to conjugate PEG to SPIONs^10^.

The long initial increase in relative absorbance for PDS0 and PDS1 eventually decreases, which originates from gravitational settling of aggregates. PDS8, in contrast to PDS0 and PDS1, shows a constant relative absorption up to 5 h. The difference between the colloidal stability of PDS1 and PDS8 suggests that there exists a threshold for sufficient surface coverage. Such observation is in agreement with a previous report^32^. Indeed, a high PEG packing density has been found to be crucial to prevent adsorption of especially small proteins^27^, which is mainly due to the formation of conformation cloud that we discussed earlier.

### Coating Influence on ROS generation

**Figure 5:**
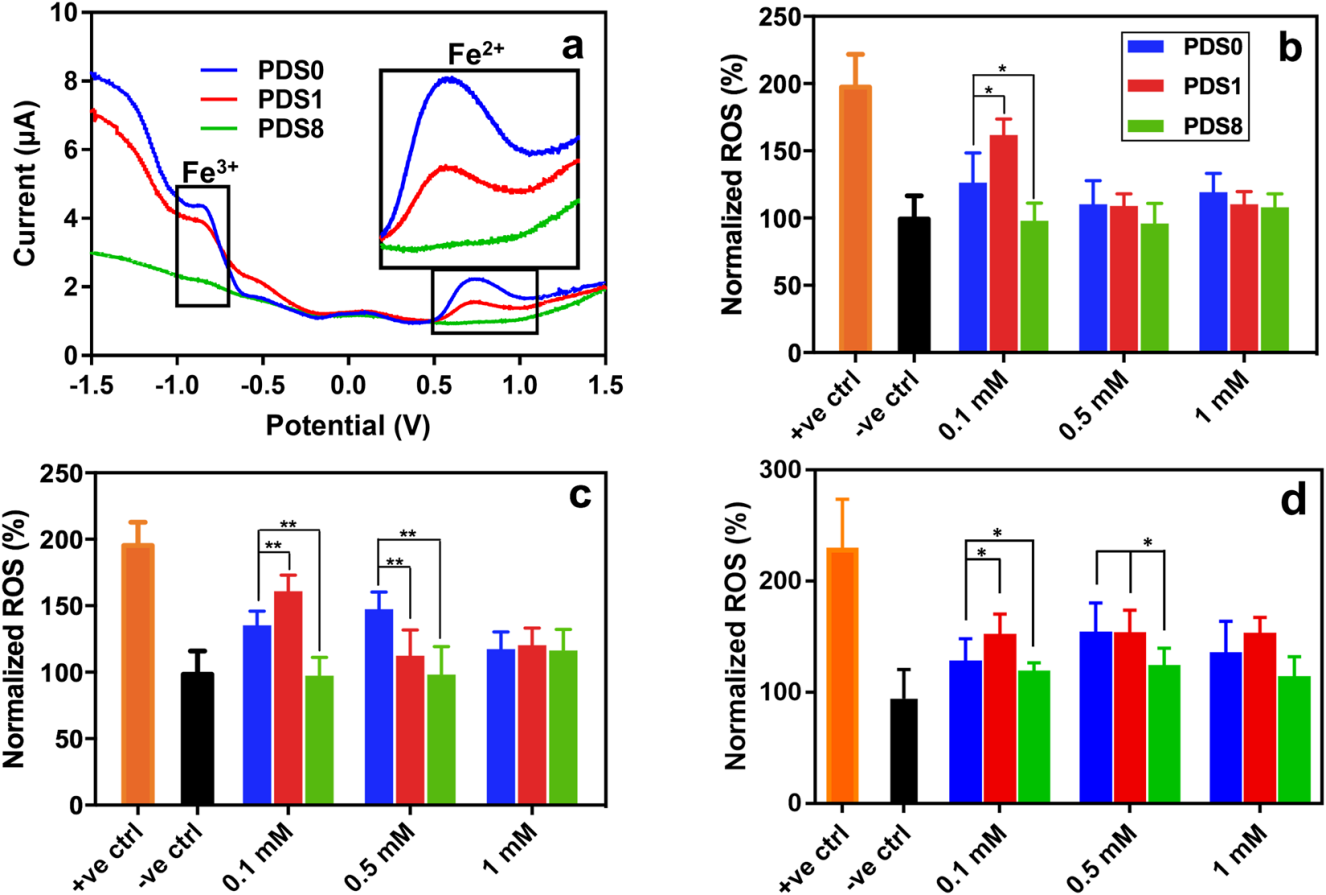
Effect of coating on Reactive Oxygen Species (ROS) generation. Anodic Spike Voltammetry analysis of NPs (**a**) shows that addition of PEG weight ratio around NPs reduces the release of Fe^2+^ and Fe^3+^. Generation of hydroxyl ion using the fluorescent dye DCFH-DA in cardiomyocytes, cardiac progenitor cells and RAW 264.7 macrophages cultured with NPs for 4 h are shown in (**b**), (**c**) and (**d**), respectively. It was revealed that incorporation of cells with PDS8 in either of the concentrations does not induce significant hydroxyl generation and is comparable with negative control. Statistical analysis was assessed against PDS0, where *, and ** denote p < 0.05, and p < 0.01, respectively. Data represent mean ± SD (n = 3).

We sought to elucidate the effect of different coatings developed in this study on the Fe ion leaching, using Anodic Spike Voltammetry (ASV) responses of NPs. The stripping peak at −0.84 and +0.75 volt in Fig. 5a shows the concentration of free Fe^3+^ and Fe^2+^, respectively^33^, which is likely to originate from the leaching of Fe ions from the surface of SPIONs into the medium. By increasing the PEG weight ratio, the concentration of free Fe ions decreases to the point where the ASV cannot sense free Fe ions for PDS8.

To further investigate the effect of PEG on controlling the generation of intracellular ROS, DCFH-DA fluorescent dye was employed. Following by cardiomyocyte, cardiac progenitor cells (CPC), and RAW 264.7 macrophages exposure to three concentrations of NPs (0.1, 0.5, and 1 mM) for 4 h, DCF fluorescence level was measured as the relative ratio of negative control (untreated cells). Figs 5b,c and d demonstrate the generation of hydroxyl using the fluorescent dye DCFH-DA in cardiomyocytes, CPCs, and RAW 264.7 macrophages, respectively. We then picked the phagocytotic cell line (RAW 264.7 macrophages) and performed cell uptake analysis (Fig. S7). Moreover, a control group was also tested for ROS generation by adding deferoxamine, a well-known iron chelator (Fig. S8). Addition of 100 µM deferoxamine reduced the ROS generation in cells exposed to PDS0 and PDS1 throughout all tested concentrations. Albeit, ROS generation mediated by PDS8 was not influenced by the presence of deferoxamine, probably due to comparable ROS generations of cells exposed to PDS8 with negative control. These results in their totality suggest that an optimized PEGylation could reduce the ROS generation likely due to less NPs biodegradation and/or reduced surface availability of NPs for catalytic activity.

### Biodistribution and pharmacokinetics

**Figure 6:**
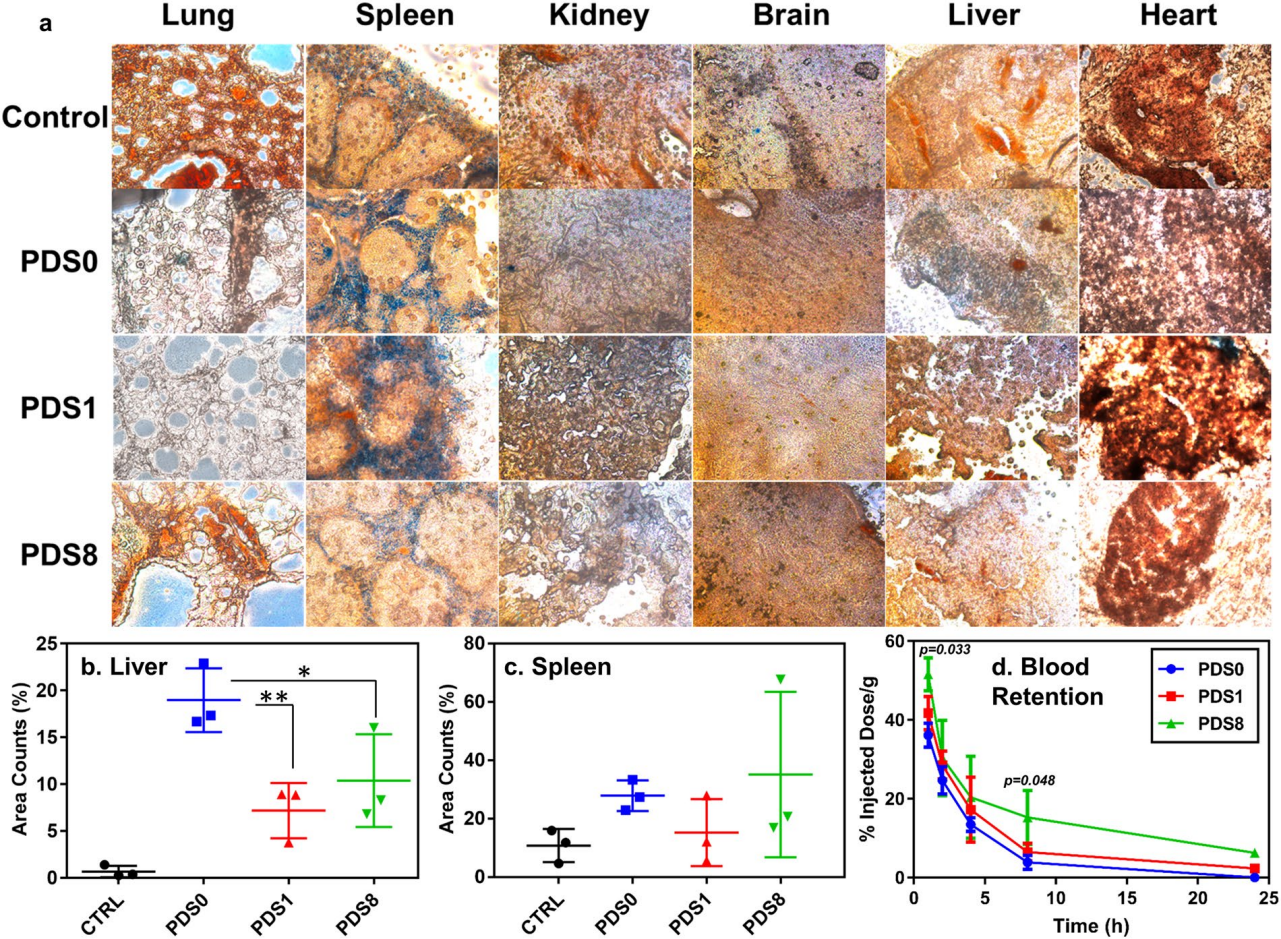
Biodistribution and blood retention analyses 24 h post injection. (**a**) 40 μm tissue sections from different organs were stained by Prussian blue. Control group was injected with PBS. Dark blue spots indicate the presence of Fe. Note that natural ferric ions exist in spleen. Figures are taken by 10X magnification. Image quantification results for (**b**) liver and (**c**) spleen. (n = 3) (**d**) Blood retentions of samples over 24-h post injection. All the mice were administered intravenously with 2 mg/mL dose of NPs. Data represent mean ± SD (n = 3) of injected dose percentage against time. Statistical analysis was assessed against PDS0.

To evaluate the clinical relevancy, we performed the qualitative analysis to find out the fate of NPs in a mouse model. Histological analysis of tissue sections stained with Prussian blue are shown in Fig. 6a. Results ascertained that the fate of NPs 24 h post injection is mainly marginal zones around the white pulp region of the spleen, which is in agreement with previous reports^34^. Figs 6b,c shows quantified results for spleen and liver of mice (n = 3).

To assess whether physisorbed PEG influences stealth properties of NPs, *in vivo* pharmacokinetic study was conducted. Mice were intravenously (*i.v*.) injected with 100 μL of 2 mg/mL NPs (core weight). Blood was withdrawn at 5 different time points within 24 h post injection, and Fe concentration was measured via ICP-OES. 100 μL of PBS was injected to mice as control to measure the intrinsic iron content of the blood, owing to the presence of elemental iron in the hemoglobin of blood. Fig. 6d indicates that PEG incorporation creates enhancement of blood retention over other samples. As we expected, PDS0 demonstrated the least circulation half-life, where they rapidly cleared out of blood circulation, and could not be detected after 8 h of injection.

## Discussion

Despite their recognized biomedical benefits, safety concerns have impaired the clinical translation of SPIONs. Unprotected surface of SPIONs mediate the generation of hydroxyl radical through catalytic activity^15^. In addition, biodegradation of SPIONs in endosomal environment generates oxidative stress known to be the major source of SPIONs toxicity^11^. Commercial classes of SPIONs are generally coated with dextran, and some reports have shown that this formulation is highly prone to biodegradation^14^. In the present study, we hypothesized that a double layer polymeric corona rather than a single layer could limit the release of Fe ions from SPIONs and/or limits the accessibility of molecules to the catalytic surface of SPIONs at the core. Therefore, SPIONs were first coated with a dextran layer following by immobilization of different amount of exterior PEG layer. Extensive physicochemical analyses confirmed the presence of both layers around SPIONs.

Colloidal stability assessments showed rapid coagulation of dextran coated SPIONs due to the weak physisorption of the dextran to the SPIONs^27^. Other reports also have indicated that dextran coatings on iron oxide NPs are prone to detachment, coagulation, and precipitation^35^. We observed that addition of PEG layer declined the coagulation kinetic. For instance, the sudden onset of coagulation for PDS1 shifts to about 4 h comparing to 1.5 h for PDS0 (Fig. 4c). This offers that the presence of the outermost PEG layer protects NPs from fast agglomeration, likely due to reduction of biomolecules access to the core of NPs. Moreover, PEG reduces undesired interaction with plasma proteins, resulting in superior colloidal stability^36^.

Other noticeable fact in colloidal stability study is that increase in absorbance appeared in CCM, but not the aqueous medium. This elevation occurs because of two opposing effects: (i) the reduction in UV absorption due to particle sedimentation, and (ii) increase in UV absorption arising from particles aggregation. Particles exposed to CCM experience a rapid agglomeration kinetic that even the decrease in UV absorption cannot compensate the absorption enhancement^30^.

As we hypothesized in this work, a proper coating around the surface of NPs could minimize the release of Fe ions. Our electrochemical analysis (Fig. 5a) revealed that certain ratio of PEG/dextran around SPIONs (PDS8) may efficiently prevent the leaching of Fe^2+^ and Fe^3+^ ions. To further validate the protective effect of PDS8 formulation on masking the core of NPs, we tested the mediating effects of NPs on cells ROS generation. Noteworthy that both ions could generate ROS^37–39^. Fe^3+^ can turn into Fe^2+^, and Fe^2+^ can react with hydrogen peroxide and the oxygen produced by the mitochondria to generate highly reactive hydroxyl ions (^•^OH) via Fenton or Haber-Weiss reactions^11,35^. ^•^OH then creates toxicity by attacking single or double strands in DNA, and reacting with lipid and polysaccharides.

Due to their different behavior towards ROS, cardiomyocytes and CPCs were chosen to study NPs ROS generation. While cardiomyocytes have peroxidase-like activity^40,41^ that limits their sensitivity to ROS, CPCs are susceptible to elevated levels of ROS^42,43^. In addition, a phagocytotic cell line (RAW 264.7 macrophage) was also assessed. Below 1 mM NPs concentration, PDS8 formulation considerably reduced ROS generation for CPCs and RAW 264.7 compared to PDS0 (Fig. 5c,d). For cardiomyocytes, however, PDS8 did not induce a notable change in ROS level for NPs concentration higher than 0.1 mM. This is likely due to the fact that SPIONs could even reduce the intracellular ROS concentration of cardiomyocytes through peroxidase-like activity of cardiomyocytes^40^. The reason behind insignificant ROS generation at higher NPs concentration is not clear to us. Albeit, a recent work also reported a significant ROS production at intermediate concentrations of SPIONs, but not higher concentrations^44^. In their work, the maximum ROS concentration was observed at 10 and 50 µg/ml of SPIONs, but not at higher concentrations (i.e. 150, 200, and 250 µg/ml). The concentrations of our samples are 0.1, 0.5, and 1 mM, which corresponds to 20, 100, and 200 µg/ml, respectively. Therefore, we postulate that the less ROS production at SPIONs concentration of 1 mM (200 µg/ml) is probably owing to the antioxidant effects of cells^44^.

We also found that the amount of PEGylation influences the ROS production, where the lower amount of PEG (PDS1) at 0.1 mM SPIONs surprisingly shows negative impact and enhances the ROS production. This could be due to larger diameters of PDS1, putting them in a suitable range for maximum cell uptake^45^ (Fig. S7, Supplementary Table 3). At >0.1 mM concentrations the cell uptake of PEGylated NPs was higher but not significant, which agrees with previous reports that there is a plateau for cellular uptake of NPs as a function of concentration^46^. In addition, mild PEGylation is probably not sufficient to control Fe^2+^ and Fe^3+^ ion release (Fig. 5a). Thus, the combination of higher uptake and incomplete surface protection are two possible reasons that PDS1 demonstrates higher ROS generation at low concentrations.

To further asses the correlation between Fe ion release and ROS generation, we hypothesized that chelating Fe ions with an iron chelator in the cell culture may reduce the ROS production of our NPs. We found that 100 µM deferoxamine remarkably reduces the ROS production in PDS0 and PDS1 samples (Fig. S8) after 4 h on NPs incubation with RAW 264.7 cells. Deferoxamine did not reduce the ROS generation in PDS8 samples, which is likely due to the sufficient blocking of Fe ion release by PDS8 formulation comparable to negative control.

Here we studied the influence of surface masking on ROS generation over a short-term basis (4 h incubation). Longer surface masking of SPIONs could be achieved with inert inorganic moieties such as gold^47^ and silica^48^. For instance, it has been shown that a protective gold layer plays a shielding effect and prevents the massive intracellular biodegradation of SPIONs over one month^47^.

We observed that PDS0 tended to accumulate in liver more than PDS1 and PDS8 24 h following *i.v*. injection (Fig. 6a,b). One possible explanation is that PEGylation reduces the phagocytosis of NPs by liver kupffer cells, providing more time for the macrophages of spleen to remove NPs from the blood^49^. Marginal zones around the white pulp region of the spleen were the major destination of injected NPs. Interestingly, we found a heterogenous sub-tissue distribution of PEGylated NPs in the spleen specially for PDS8 (Fig. 6c). We also could not observe any iron accumulation in brain, heart, and lung, which is consistent with observations reported elsewhere^50^. While these observations provide an initial proof of concept, quantitative analysis (e.g. ICP-MS) should be conducted in the future studies for accurate comparison of biodistributions. Finally, PDS8 showed enhanced blood retention time (15.28% ± 5.31, p = 0.048) compared to PDS0 (3.88% ± 1.78) 8 hours following administration (Fig. 6d). A recent study reported a half-life of 155 mins on covalently bonded PEG (5 kDa) on SPIONs^51^, while the half-life of PDS8 in our study is less than 1 h, which is likely due to the weak attachment of PEG to NPs.

## Conclusion

In summary, we immobilized a PEG layer around dextran-coated SPIONs to fabricate a double layer protection around the core NPs. The presence and amount of external PEG layer enhanced colloidal stability, and influenced ROS generation, biodistribution and pharmacokinetic of NPs. Optimum amount of PEG layer reduced the ROS generation mediated by SPIONs, likely due to less biodegradation and/or reduced surface availability of NPs for catalytic activity. Addition of deferoxamine, an iron chelator, also reduced the ROS generation by SPIONs. Moreover, NPs concentration and cell type were two critical factors determining the ROS generation profile of NPs. While results from 24 h NPs injection indicated that PEG immobilization did not remarkably alter the blood retention, biodistribution in liver was reduced and NPs distribution in spleen demonstrated a more heterogenous profile. Our work suggests that sufficient surface masking or administration of deferoxamine could be beneficial in designing and clinical translation of future biomedical SPIONs.

## Methods

### Materials

All the chemical reagents were of analytical grade and used without further purification, unless otherwise noted. The detailed reagents were included in the supplementary materials.

### Preparation of PEG/dextran coated iron oxide NPs

Dextran coated SPIONs (PDS0) were synthesized via coprecipitation method as reported elsewhere^52^ with some modification. The detailed procedures were included in the supplementary materials. We employed a very facile technique to physically immobilize PEG around dextran coated SPIONs via adding PEG to other two batches with the following weight ratios of PEG/SPION: 1/1, and 8/1 (samples PDS1, and PDS8, respectively) and stirred for 24 hours via magnetic stirrer at 1000 rpm. Next, all samples were separated over a strong NdFeB magnet and the supernatant was decanted. Finally, obtained NPs underwent additional sonication step to breakdown any agglomerated clusters formed during magnetic decantation to be used as suspension for colloidal evaluations.

### Characterization methods

Physicochemical properties of NPs were characterized using various methods, and the detailed procedures were included in the supplementary materials.

### Colloidal stability studies

Colloidal stability analyses were conducted based on optical absorbance density of the prepared suspensions measured via a UV-VIS spectrophotometer (Agilent Cary 6000i UV/Vis/NIR, USA). Samples were diluted with DI H_2_O and their concentrations were adjusted to be 50 μM. Colloidal stability was assessed based relative absorption (A) over the initial absorbance value (A_0_). Experiments in CCM were performed by pipetting 100 μL of 50 μM samples to 3.9 ml of the RPMI 1640 cell culture medium supplemented with 10% FBS. Subsequently, absorption through 1 cm light pathway square glass cuvette was recorded (λ = 550 nm). Measurements were carried out in triplicate. The obtained optical densities were plotted as A/A_0_ against time.

### Elechtrochemical analysis

Anodic Spike Voltammetry (ASV) was conducted using the microfabricated three electrode system connected to Interface 1000 potentiostat (Gamry Instruments). NPs concentration were adjusted to be 100 µM. The three electrode system consisted of microfabricated Pt working electrode, Pt counter electrode and a Ag/AgCl reference electrode. Using the known standard reduction potential of the reference electrode, the potential applied to the working electrode by means of the potentiostat was controlled and regulated and the redox reactions were studied by measurement of the current. In this experiment, the Pt working electrode was held at a constant potential of -1.5 V for 120 s and then scanned towards 1.5 V vs. the Ag/AgCl reference electrode^53^. The other important parameters for this experiment includes a frequency of 25 Hz and a pulse size of 25 mV.

The iron oxide NPs are accumulated around the reference electrode when held at a constant potential while gaining electrons. Subsequently, when the potential is scanned towards 1.5 V, the iron oxide NPs return into the solution and the measured current corresponds to their concentration on the surface.

### Determination of reactive oxygen species

RAW 264.7 macrophages, CPCs and cardiomyocytes were plated in a 96 well plate at a density of 10,000 cells per well. RAW 264.7 macrophages were cultured in DMEM medium with 10% FBS with 1% antibiotic. CPCs were cultured in DMEM/F12 medium with embryonic stem cell FBS and plated on a 0.2% gelatin-coated 96-well plate. Cardiomyocytes were cultured in RPMI with B27 supplement and plated on a matrigel coated 96 well plate. The cells were cultured at 37 °C in a 5% CO_2_ incubator. Cardiomyocytes were less than 30 days of age post-differentiation. Cells were cultured in 100 μl medium for each well for 24 h, following by addition of the medium containing three different concentrations of SPIONs i.e. 0.1, 0.5, and 1 mM (The amount of iron was confirmed via atomic absorption). The control wells contained CCM without SPIONs. Another negative control was tested by adding 100 μM of deferoxamine to SPIONs containing batches. Tests were conducted in triplicate, and all samples including the control, were placed in five wells to provide statistically reliable results. The intracellular amount of ROS is a critical biomarker for oxidative stress, and elevated level of ROS is generally an indication for increased oxidative stress. Generation of ROS was assessed via the fluorescent dye DCFH-DA, which is a nonpolar compound converted by cellular estrases to fluorescent 2′,7′-Dichlorofluorescin (DCF). Intracellular ROS turns a non-fluorescent DCFH-DA to fluorescent DCF. After treatment of cells with NPs for 4 h, cells were washed with PBS and loaded with DCFH-DA (20 μM) and incubated for 30 minutes at 37 °C, and 5% CO_2_ atmosphere. Hydrogen peroxide-treated cells (100 μM H_2_O_2_) and untreated cells were used as positive and negative controls, respectively. In addition, a control group was also tested by adding 100 µM of deferoxamine, a well-known iron chelator. Cells were then washed twice with PBS (to prevent interference) and analyzed using the FlexStation 3 multiplate reader at an excitation wavelength of 485 nm and emission wavelength of 535 nm. Data are expressed as fluorescence ratio compared with the relevant negative controls.

### Cell uptake, *in vivo* pharmacokinetics, and biodistribution studies

The detailed procedures were included in the supplementary materials. Seven-week old CD-1 female mice were used in this study. Animal handling, surveillance, and experimentation were performed by *Bayside BioSciences Inc*. in accordance with and approval from the Stanford University Administrative Panel on Laboratory Animal Care (protocol no. 2016-021).

### Statistical analysis

Statistical differences were analyzed by two-tailed t-test using Microsoft Excel software with analysis tool pack add-ins (Microsoft Inc., USA). Probabilities were marked as p < 0.05 (*), p < 0.01 (**), and *n.s*. denotes as non-significant in each figure.

### Data availability

All data generated or analysed during this study are included in this published article and its Supplementary Information files and available from the corresponding author on reasonable request.

## Electronic supplementary material

Supplementary Information
