# Supplementary material for: PEG/Dextran Double Layer Influences Fe Ion Release and Colloidal Stability of Iron Oxide Nanoparticles
Source: Sci Rep. 2018 Mar 9;8:4286. doi: 10.1038/s41598-018-22644-8 (PMC5844897; doi:10.1038/s41598-018-22644-8)
Supplement: Supplementary file 1 — Supplementary Information [file 41598_2018_22644_MOESM1_ESM.docx]

**Supporting Information**

**PEG/Dextran Double Layer Influences Fe Ion Release and Colloidal Stability of Iron Oxide Nanoparticles**

M. Rezaa Mohammadi^a^, [Andrey V. Malkovskiy](http://www.sciencedirect.com/science/article/pii/S0167814016344139)^a^, Preetha Jothimuthu^a^, Kwang-Min Kim^a^, Mansi Parekh^a^, Mohammed Inayathullah^a^, Yan Zhuge^c^, Jayakumar Rajadas^a,b*^

^a^Biomaterials and Advanced Drug Delivery Laboratory, School of Medicine, Stanford University, CA 94305, USA

^b^Division of Cardiovascular Medicine, Department of Medicine, Stanford University School of Medicine, Stanford, CA 94305, USA

^c^Department of Radiology, School of Medicine, Stanford University, CA 94305, USA

Correspondence to:

Stanford University 1050 Arastradero Road, Room A148, Palo Alto, CA 94304, USA

Tel.: 650 724 6806

Fax: 650 721 4651

E-mail address: jayraja@stanford.edu

*Materials*

All the chemical reagents were of analytical grade and used without further purification, unless otherwise noted. Iron (III) chloride hexahydrate (FeCl_3_·6H_2_O), iron (II) chloride tetrahydrate (FeCl_2_·4H_2_O), sodium hydroxide (NaOH), hydrochloric acid, (HCl, 35 Wt%), nitric acid (HNO_3_, 63 Wt%), PEG (Mw=6 kDa), dextran (Mw=40 kDa), KBr, potassium ferrocyanide, trihydrate, and 2′,7′-Dichlorohydrofluorescin diacetate (DCFH-DA) were all purchased from the Sigma-Aldrich (St. Louis, USA). O.C.T (Tissue-Plus®, Fisher HealthCare), Slide-A-Lyzer® dialysis cassette (100 kDa MWCO), and deferoxamine were purchased from ThermoFisher Scientific, RPMI 1640, and DMEM Cell Culture Medium, Penicillin, and Fetal Bovine Serum (FBS) were purchased from Invitrogen (Massachusetts, USA). 1X Phosphate buffered saline pH 7.4 (PBS) was prepared by standard protocols. Deionized water (DI H_2_O) (> 18.2 MΩ cm, Milli-Q Academic, Millipore) and high purity N_2_ were also used in experiments*.*

*Preparation of dextran coated iron oxide NPs (Sample PDS0)*

Briefly, 1 mmol (0.198 g) FeCl_2_.4H_2_O and 2 mmol (0.540 g) FeCl_3_.6H_2_O were added into a reactor containing 100 ml DI H_2_O and 5 mg dextran. Magnetic stirring was continued for 30 minutes to ensure the proper dissolving of all reagents. The resulted solution was ultrasonicated (Biologics, Inc., USA) for 30 min under ultra-pure N_2_ gas atmosphere, and the power was adjusted to 30 W in the three-necked round bottom glass. Gradually, salt solution temperature was risen to 80 °C using hot-plate apparatus. Meanwhile, 25 mM NaOH solution was purged with N_2_ gas for 10 min to remove any dissolved oxygen, and quickly dropped into the solution. Probe sonication was stopped before dropping the NaOH solution. Black precipitates of dark suspension were then washed three times over strong NdFeB magnet, followed by overnight dialysis against PBS using Slide-A-Lyzer® with 50 kDa MWCO. Subsequently, the NPs were washed 3 times and dispersed in 100 mL DI H_2_O by using a bath ultrasonic (Branson 2510, USA) for 60 minutes. Note that to obtain bare SPIONs, we synthesized another batch with exactly same procedure except adding dextran and PEG. We divided the dextran coated SPIONs into 3 different sub-batches. One sub-batch named PDS0, which represents dextran coated SPION without further reaction. Two other samples were PDS1: a physically PEGylated PDS0 with 1:1 PEG:SPION (w/w), and PDS8: a physically PEGylated PDS0 with 8:1 PEG:SPION (w/w).

*Characterization Methods*

The phase identification of synthesized NPs was performed by X-ray diffraction (XRD, Bruker D8 Venture, USA) method using a Cu-Kα radiation (λ= 1.5406 Å).

Amount of adsorbed PEG layer on the surface of NPs was evaluated via thermogravimetric analysis (TGA, Perkin-Elmer TGA7, USA) scanning from 25 °C to 700 °C at 10 °C/min heating rate in the presence of N_2_ gas. In each set of experiment 100 mg freeze dried samples were employed.

Magnetization of samples was measured via a vibrating sample magnetometer (VSM, Lake Shore, Inc.) at 300 K. Sample magnetization was recorded as a function of the applied magnetic field. Field strength was varied in the range of ±10 kOe at an operating frequency of 100 Hz.

Fourier transform infrared spectroscopy (FTIR, Bruker Vertex, USA) was carried out to assess the surface bands of NPs. Dried NPs were mixed with KBr powder, and then were pressed to form pellets. Raman spectroscopy was conducted to further analyze the double layer polymeric coating. The Raman measurements have been performed using backscattering geometry on SPION and pure components, dried in air on titanium foil. The illumination source was a 473 nm Cobolt Blues™ continuous wave diode-pumped solid-state laser brought to the sample by a Mitutoyo long working distance objective (100X, 0.7 NA). Laser power at sample was 1.08 mW, as measured by Coherent LaserCheck™. The spectra were recorded for 600 s or sometimes longer, until sufficient amount of counts was accumulated or the sample started showing signs of degradation. Raman spectra were aquired in with an Andor CCD peltier-cooled camera (as part of a NTEGRA Spectra system) with a 600 grating from solid samples. Each spectrum was monitored for signs of sample degradation and discarded if any were observed. Raman spectra were baseline corrected to account for fluorescent background.

NPs size and morphology were investigated via field emission scanning electron microscope (FESEM, Zeiss Sigma, Germany) with Schottky Field Emission source and GEMINI electron optical column according to a reported method [1]. The core size and morphology of NPs were studied by transmission electron microscopy (TEM, JEOL, JEM1400, USA) under 120 kV with a LaB_6_ emitter providing and digital image acquisition equipped with a Gatan Orius 10.7-megapixel CCD camera. TEM samples were prepared by diluting samples with DI H_2_O (10% v/v), following by placing a droplet of the diluted suspension on a carbon-coated copper grid. Image analysis was performed by ImageJ software. To further analyze the NPs, Atomic Force Microscopy (AFM) was conducted. AFM imaging was performed with NX-10 AFM from Park Systems in non-contact mode using standard commercial cantilevers from Micromasch® with less than 30% oscillation damping. Polished Si wafers from VWR were used as sample substrates. Data analysis of AFM images was done in XEI™ (v. 1.8.1.Build214).

Dynamic Light Scattering (DLS) measurements were performed using Brookhaven 90 plus DLS nanosizer (Brookhaven Instruments Corporation, Holtsville, USA) according to a published protocol [2]. Samples were diluted to 50 μM (confirmed by atomic absorption spectroscopy) and were kept at 4 °C throughout the study.

*Cell uptake, in vivo pharmacokinetics, and biodistribution studies*

RAW 264.7 macrophages were seeded on a 96 well-plate (10,000/well) one day before adding the NPs. NPs were each added at a final concentration of 0.1, 0.5 and 1 mM to cell culture media. The following day, old medium was aspirated off, and cell were washed with 1X PBS twice. The fresh medium containing NPs were added to cells (100 μl/well). Cells were then treated with 100 μL of 10 mM HCl following by incubation at 60 °C for 2 h. Fe concentration was measured via ICP-OES (ThermoFisher Scientific ICAP 6300, Duo View Spectrometer). Mice were injected with 100 μL of 2.0 mg/mL NPs through the tail-vein, and control mice were injected with 100 μL PBS. The tail was warmed with a lamp in order to dilate the vessels, and mice were anesthetized using 5% isoflurane. Respiration rate was monitored and maintained between 100 and 150 breaths/min. Following by 24-h of injection, mice were euthanized by cervical dislocation and their organs were harvested and fixed in 4% formaldehyde solution. Organs were embedded in O.C.T formulation (Fisher Scientific, USA) for histological analysis, using cryosectioning (Leica, CM1950 Cryostat). Liver, spleen, kidneys, heart, lungs and brain were collected, and sectioned to slices with 40 μm thickness. Tissue sections were stained for iron using Prussian blue, and images were visualized under light microscopy at 10X magnification (Leica Microscope, Leica DM 4000B; Leica Microsystems) and photographed using the Leica DFC 500 camera (Leica, Allendale, USA). For pharmacokinetic studies, 50 μL blood was withdrawn at 5 different time points within 24-h post injection (1, 2, 4, 8, and 24 h), and Fe concentration was measured via ICP-OES (ThermoFisher Scientific ICAP 6300, Duo View Spectrometer). 100 μL of PBS was injected to control mice to measure the intrinsic iron content of the blood, owing to the fact that blood hemoglobin contains elemental iron.

**Supplementary Table 1.** Descriptive Raman peaks and their corresponding bands and vibrational state

| **Raman frequency, cm^-1^** | **Assignment** |
| --- | --- |
| 845 | CH_2_ rocking |
| 860 | CH_2_ rocking |
| 1068 | CO stretch |
| 1130 | CC stretch |
| 1143 | CO stretch |
| 1238 | CH_2_ twist |
| 1284 | CH_2_ twist |
| 1365 | CH_2_ wag |
| 1400 | CH_2_ wag |
| 1447 | CH_2_ bending |
| 1484 | CH_2_ bending |

**Supplementary Table 2.** Saturation magnetization (Ms) values for samples

| **Sample** | **Ms, emu/g** |
| --- | --- |
| PDS0 | 57.0 |
| PDS1 | 56.3 |
| PDS8 | 51.4 |

**Supplementary Table 3.** Size analysis of NPs. Note that large scale aggregates were eliminated from size analyses

|  | **PDS0** | **PDS1** | **PDS8** |
| --- | --- | --- | --- |
| **TEM** | 6.8±1.2 | 12.4±2.7 | 16.6±4.7 |
| **SEM** | 12.3±4.6 | 33.6±8.3 | 49.6±10.9 |
| **DLS** | 32.4±8.1 | 46.2±4.9 | 53.0±7.5 |


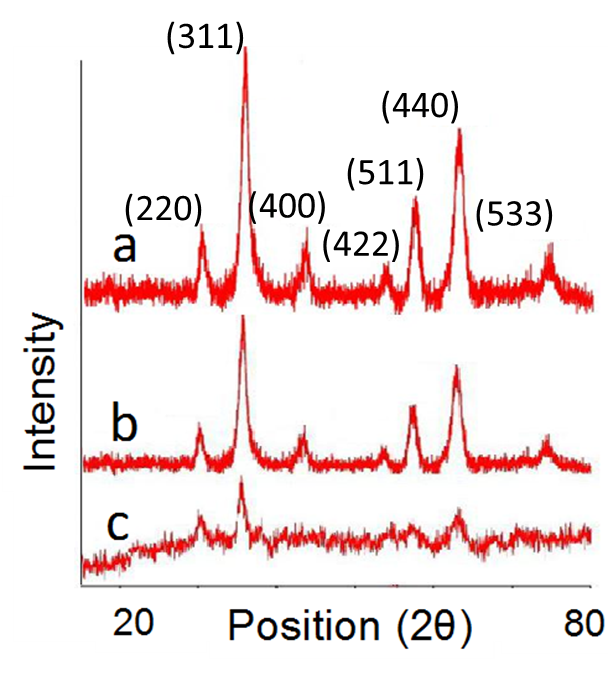


**Figure S1.** XRD pattern of (a) PDS0, (b) PDS1, and (c) PDS8 show the effect of polymeric corona on the crystalline structure of NPs. Diffraction pattern with corresponding planes are well-matched with the magnetite (Fe_3_O_4_) diffraction peaks (JCPDS card no. 19-0629), confirming their inverse spinel structure. It is clear that crystal structure of magnetite is not affected by polymeric coating, as amorphous PEG cannot interfere with X-ray diffraction of SPIONs.


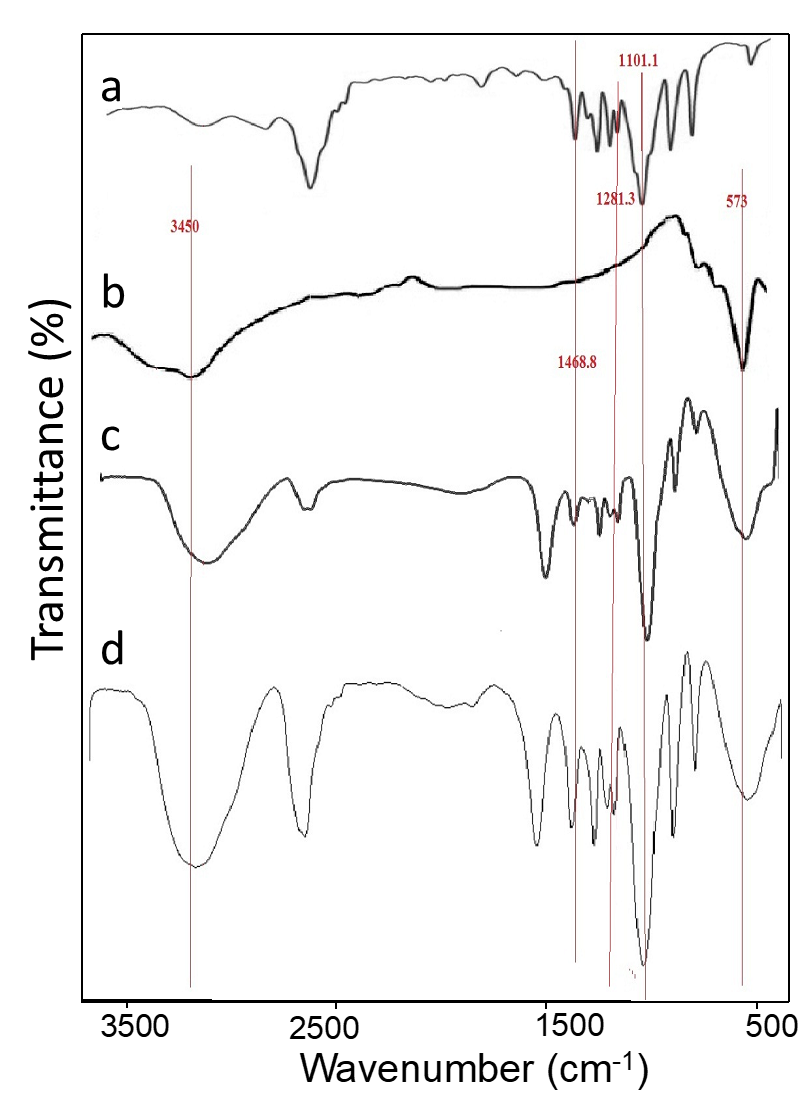


**Figure S2.** FTIR spectrum of (a) PEG, (b) SPION, (c) PDS1, and (d) PDS8 demonstrate the bond formation between NPs, dextran, and PEG. –C–O–C– ether stretch band and the vibration band (antisymmetric stretch) are appeared at 1101.1 cm^-1^ and 1349.4 cm^-1^, respectively. Absorption bands at 1281.3 cm^-1^ and 1468.8 cm^-1^ attribute to the vibration of –CH_2_. The characteristic absorbance peaks of PEG show a slight shift to lower frequencies (red shift) due to changing the environment of PEG as well as hydrogen bonding between PEG and dextran.


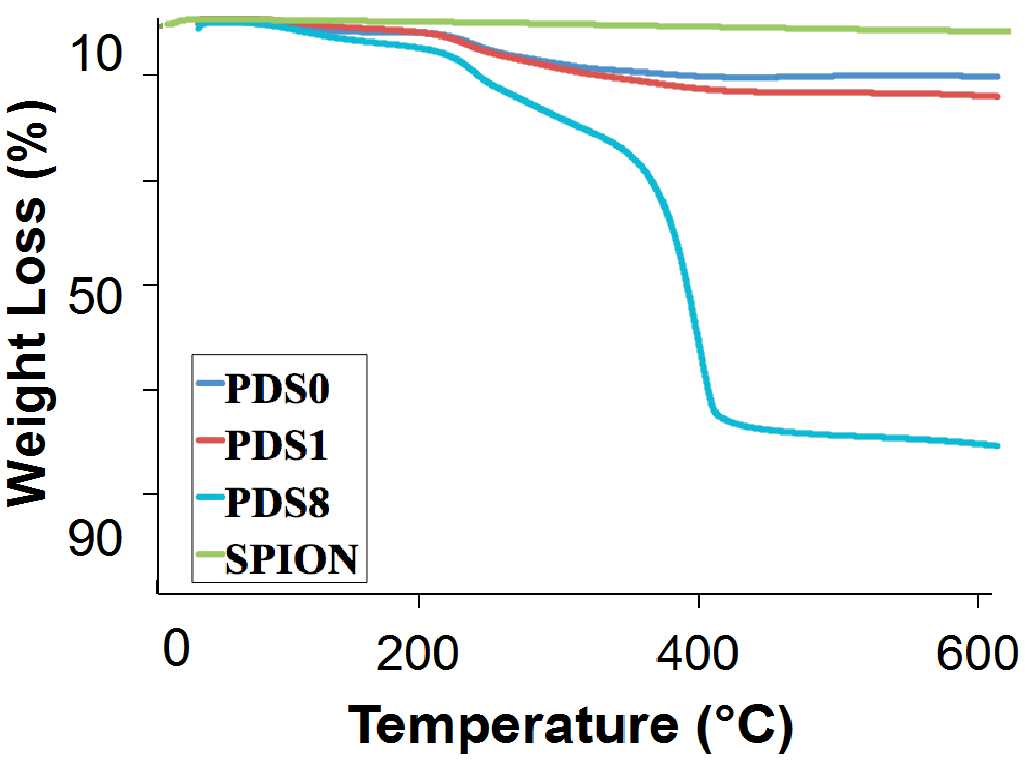


**Figure S3.** TGA analysis of SPION, PDS0, PDS1, and PDS8, illustrating the degradation profiles of samples in response to temperature.


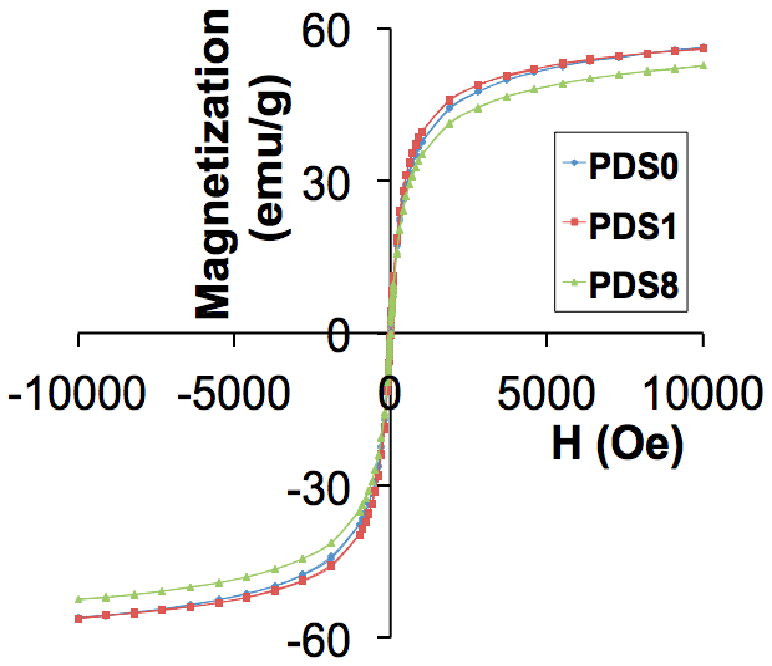


**Figure S4.** VSM analysis of PDS0, PDS1, and PDS8, demonstrating the effect of coating coronas on magnetic behavior of NPs.


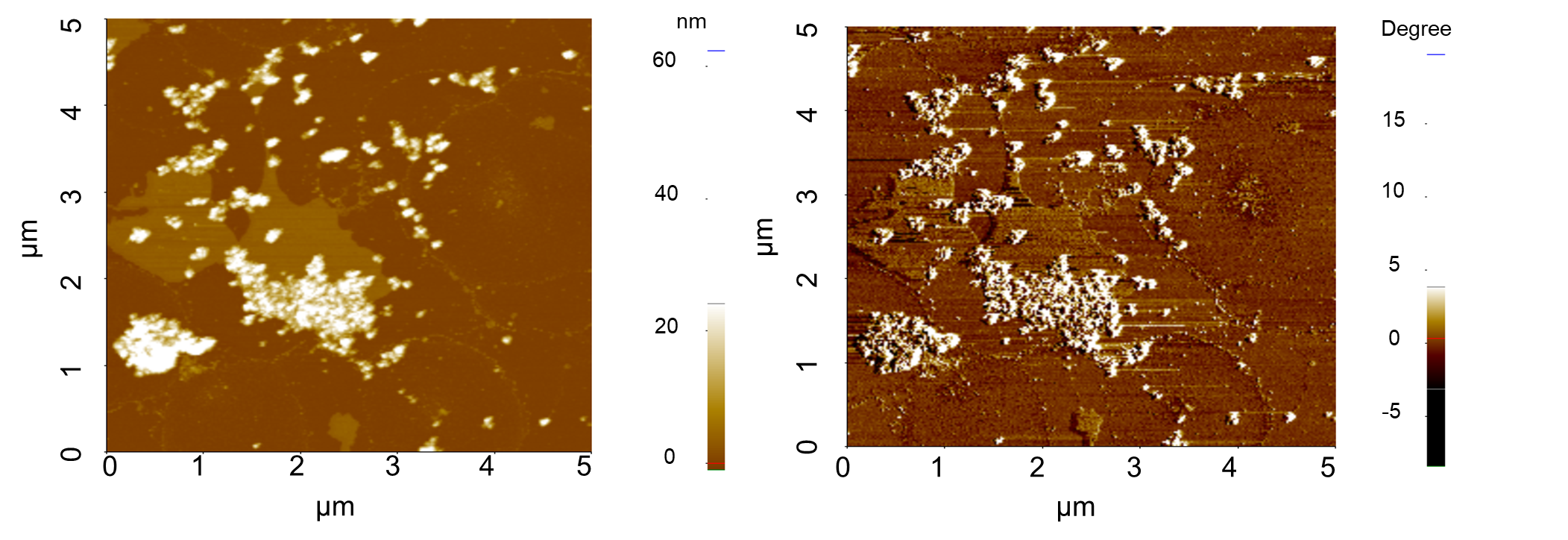


**Figure S5.** Analysis of PDS0 with 5 square micron AFM maps. Figure in the left and right represent the height and phase images, respectively. PDS0 samples demonstrate the presence of nanoscale particles as well as large aggregates.

**
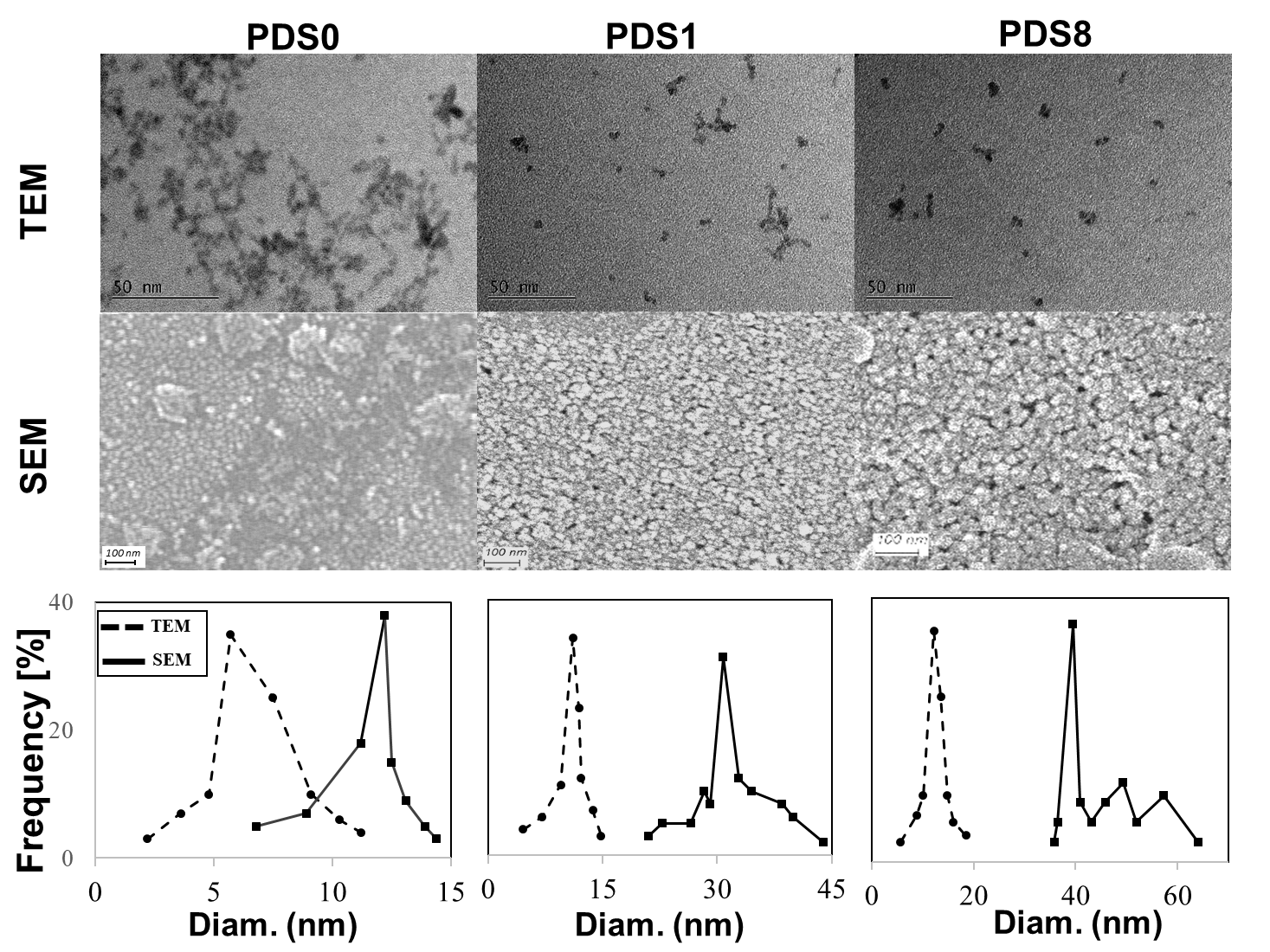
**

**Figure S6.** TEM, FESEM, and ImageJ size analysis micrographs of samples PDS0, PDS1, and PDS8. (Scale bar for TEM is 50 nm and for FESEM is 100 nm). Large aggregates were eliminated from size evaluations. Results demonstrate individual NPs as well as larger aggregates are present in PDS0. Large aggregates were still observable after PEGylation (PDS1). By addition of more PEG, large aggregates were not observable in large scale, however, small colonies were appeared (PDS8).


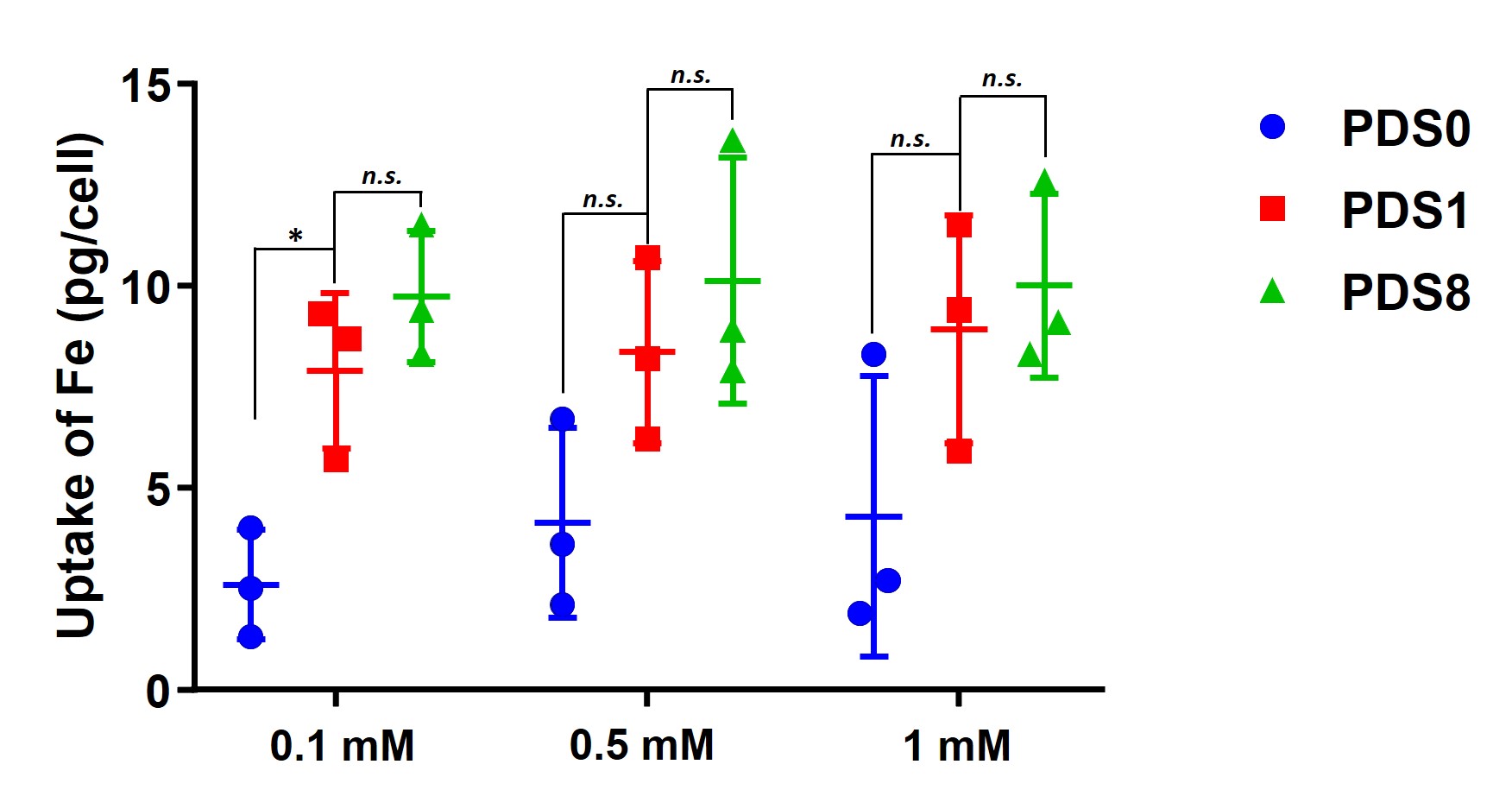


**Figure S7.** Cell uptake quantification 4 h following by incubation of NPs. Statistical analysis for PDS1 was assessed against PDS0, and for PDS8 was assessed against PDS1. * and *n.s.* denote p < 0.05, and non-significant, respectively. Data represent mean ± SD (n=3)


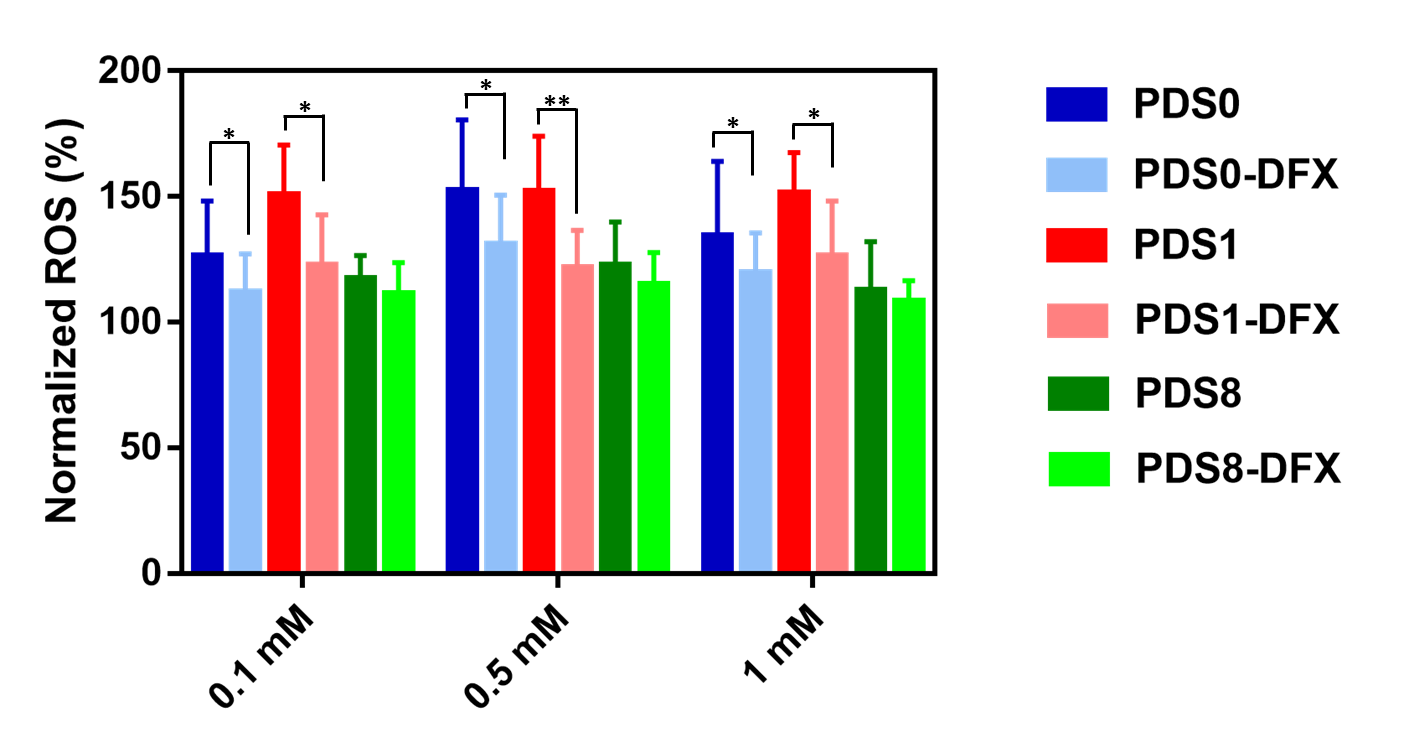


**Figure S8.** Effect of deferoxamine (DFX), an iron chelator, on Reactive Oxygen Species (ROS) generation induced by NPs. Generation of hydroxyl ion using the fluorescent dye DCFH-DA in RAW 264.7 macrophages was evaluated in the presence and absence of DFX. Incorporation 100 µM DFX significantly reduces the ROS generation caused by NPs. Insignificant difference of ROS generation in the presence or absence of DFX for PDS8 sample is likely due to the efficient blocking of Fe ion release. Statistical analysis was assessed between NPs and NPs cultured with 100 µM DFX, where *, and ** denote p < 0.05, and p<0.01, respectively. Data represent mean ± SD (n=3)

1. Nojoomi, A., et al., *Injectable polyethylene glycol-laponite composite hydrogels as articular cartilage scaffolds with superior mechanical and rheological properties.* International Journal of Polymeric Materials and Polymeric Biomaterials, 2017. **66**(3): p. 105-114.

2. Pandey, N., et al., *Biodegradable Nanoparticles Enhanced Adhesiveness of Mussel-Like Hydrogels at Tissue Interface.* Advanced Healthcare Materials: p. 1701069-n/a.
